# Variation of Ursolic Acid Content in Eight *Ocimum* Species from Northeastern Brazil

**DOI:** 10.3390/molecules13102482

**Published:** 2008-10-14

**Authors:** M. Goretti V. Silva, Ícaro G. P. Vieira, Francisca N. P. Mendes, Irineu. L. Albuquerque, Rogério N. dos Santos, Fábio O. Silva, Selene M. Morais

**Affiliations:** 1 Departamento de Química Analítica e Fisico-Química - Departamento de Química Orgânica e Inorgânica, Universidade Federal do Ceará, 60.455-970, Fortaleza, CE, Brazil; 2 Laboratório de Química de Produtos Naturais, Universidade Estadual do Ceará, Campus do Itaperí, CEP 60.740-000, Fortaleza, CE, Brazil; 3 Parque de Desenvolvimento Tecnológico (PADETEC), 60455-970, Fortaleza, CE, Brazil; 4 Universidade de Fortaleza, 60811-905, Fortaleza, CE, Brazil

**Keywords:** Labiatae, Ursolic acid, *Ocimum*, HPLC

## Abstract

Ursolic acid is a very important compound due to its biological potential as an anti-inflammatory, trypanocidal, antirheumatic, antiviral, antioxidant and antitumoral agent. This study presents the HPLC analysis of ursolic acid (UA) content in eight different *Ocimum* species: *O. americanum* L., *O. basilicum* L, *O. basilicum* var *purpurascens* Benth, *O. basilicum* var. *minimum* L, *O. gratissimum* L, *O. micranthum* Willd, *O. selloi* Benth. and *O. tenuiflorum* L. grown in Northeastern Brazil. In these *Ocimum* species, UA was detected in different yields, with *O.* *tenuiflorum* showing the highest content (2.02%). This yield is very significant when compared with other sources of UA.

## Introduction

**Figure 1:**
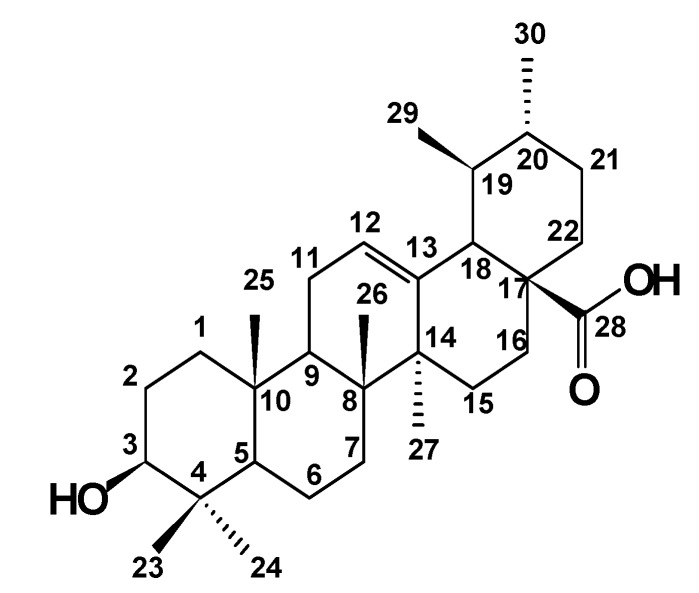
Chemical structure of ursolic acid (**1**).

Ursolic acid (3β-hydroxy-urs-12-en-28-oic acid) is an ursane type triterpene (Figure 1) found in all parts of plants, but mainly in leaves and presenting several important biological activities. These include anti-inflammatory, antioxidant and anti-tumoral properties, being effective in reducing the growth of a variety of cancer cell lines *in vitro* [1,2,3,4,5]. The anti-inflammatory activity in *Sal*v*ia officinalis* L. leaves was attributed to ursolic acid and this compound showed activity two times greater than indomethacin. Topical application of 10 μmol of UA for 20 weeks inhibited 78% of skin tumors [3, 6]. Other relevant activities such trypanocidal, antirheumatic and antiviral properties are attributed to the presence of UA in many plants [7].

*Ocimum* is one of the most important genus of the Lamiaceae family, due to the extensive use of many of its species as economically important medicinal and culinary plants. Ursolic acid was previously identified in only two species of *Ocimum*: *O. basilicum* and *O. tenuiflorum* [8, 9]. According to ethnobotanical information people from Northeastern Brazil have been using infusions of *Ocimum* species for ritualistic aromatic baths, and as a tea for treating gastro-intestinal problems and also for seasoning special foods [10]. This study presents the variation of ursolic acid content in eight species of the genus *Ocimum*: *O. americanum*, *O. basilicum*, *O. basilicum* var *purpurascen*s, *O. basilicum* var. *minimum*, *O. gratissimum*, *O. micranthum*, *O. selloi* and *O. tenuiflorum* grown in the Northeast of Brazil, as determined by HPLC analysis

### Results and Discussion

**Figure 2:**
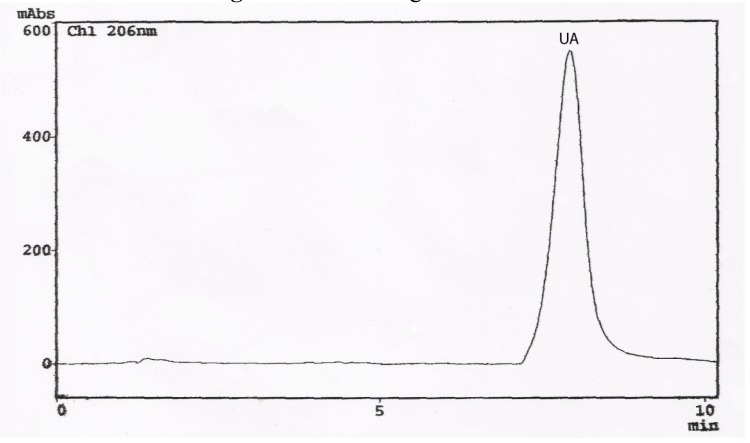
Chromatogram of UA.

**Figure 3:**
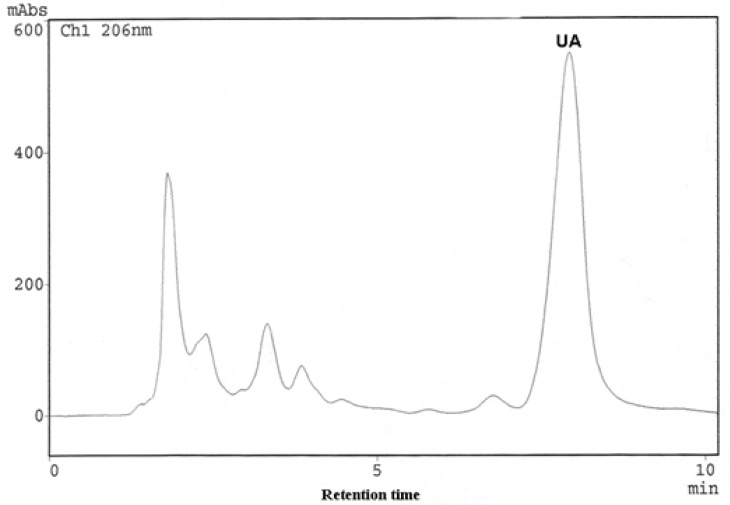
Chromatogram of a methanol extract of *O. gratissimum.*

Commercially available ursolic acid is extracted from *Rosmarinus officinalis* leaves native to Southern Europe with a yield of 1.5% (by wt.) [11]. High amounts of this triterpenoid compound had also been detected in *Plectranthus* and *Salvia* species [6, 12]. The percentage yields of UA in dried leaves of *Ocimum* species from Northeast of Brazil was evaluated by HPLC-PDA, enabling the determination of retention time (Rt) and UV-Vis spectra. The UA in the extracts was compared against an UA standard. The identity was established by overlay of the absorption spectra of UA (in each example extract) with the UA standard (Figure 2). A chromatogram of *O. gratissimum* is shown in Figure 3, where the retention time (Rt) of UA was 7.92 min. Calibration graphs for UA were constructed in the 3.60 – 72.00 μg/mL range. The regression equation of this curve and its coefficients of determination (R^2^) were calculated as follows: Y=1.3050E+06X-1.5050E+04 (R^2^=0.9999); limit of quantification 0.1 μg/mL; limit of detection 0.04 μg/mL; relative standard deviations (RSD) less then 2.0 %. The eight differents sample solutions were analyzed in the same manner, the peaks were identified by comparison of the retention time corresponding to authentic UA purified from *O. gratissimum*. Regarding the extraction efficiency, repetition of the work-up three times was deemed sufficient, since it allowed over 98,00 % extraction of the UA.

In these *Ocimum* species, UA was detected in different yields (Table 1). It was found to be less in *O.* *basilicum* var. *minimum* (0.27%) and a highest content in *O. tenuiflorum* (2.02%). This last result is very significant when compared with other sources of UA and *O. tenuiflorum* constitutes a new potential source of this important compound.

**Table 1:** Percentage yields of ursolic acid (UA) in dried leaves of *Ocimum* species from Northeast Brazil.

Species	Voucher number	% UA
***Ocimum americanum***	17.611	1.03%
***Ocimum basilicum***	18.670	0.29%
***Ocimum basilicum*** var. ***purpurascens***	18.777	0.38%
***Ocimum basilicum*** var. ***minimum***	17.611	0.27%
***Ocimum gratissimum***	18.671	1.04%
***Ocimum micranthum***	29.315	1.05%
***Ocimum selloi***	27.020	0.45%
***Ocimum tenuiflorum***	14.949	2.02%

## Experimental

### General

HPLC analysis was performed on a Shimadzu LC-10AD pump system equipped with a Shimadzu SPD-M10A photodiode array detector with the detection wavelength set at 206 nm. Melting points were determined using a Microquímica MQAPF-301 melting point apparatus and NMR spectra were recorded on a Bruker DRX 500 [500 MHz (^1^H) and 125 MHz (^13^C)] spectrometer. Chemical shifts were recorded in (ppm) relative to residual solvent (2.49 ppm for ^1^H-NMR and 39.5 ppm for ^13^C-NMR). Multiple-pulse experiments (COSY, HMQC and HMBC) were performed using the standard Bruker programs.

### Plant material

Leaves of the eight *Ocimum* species were collected in April 2005 from the Francisco José de Abreu Matos Medicinal and Aromatic Plants Garden of the Federal University of Ceará (UFC). Voucher specimens were deposited in the Prisco Bezerra Herbarium of the UFC under numbers presented in Table 1.

### Extraction and Purification

The air-dried leaves of *O. gratissimum* (4.1 kg) were extracted with ethanol and the solvent was removed under reduced pressure given a solid which was submitted to a chromatographic silica gel column, sequentially eluted with hexane, dichloromethane, ethyl acetate and methanol. The ethyl acetate fraction was chromatographed on a silica gel column to yield ursolic acid (35.2 mg) which was identified by melting point and ^1^H- and ^13^C-NMR spectroscopy including a 2D sequence, comparing the UA assignments with literature data [13]. Quantification of UA in the *Ocimum* species was carried out by HPLC: leaves (5 g) were dried using a microwave oven for nine minutes (three times) then they were powdered and extracted with methanol (125 mL) using a Soxhlet apparatus for three hours. Methanol extracts were concentrated and the extracts were diluted with methanol to 10 mL. The afforded solution was filtered through a 0.45 μm syringe filter prior to HPLC use.

*Ursolic acid (3β-hydroxy-urs-12-en-28-oic acid)* (**1**): White powder, ^1^H-NMR (DMSO-d_6_) ppm: 5.14 (1H, dd, *J* = 13.7; 3.5 Hz; H-12); 3.01 (1H, dd, *J* = 5.2; 9.5 Hz, H-3); 2.12 (1H, d; *J* = 11.1 Hz, H-18); 1.92 (2H, dd, *J* = 13.7; 3.5 Hz, H-11); 1.53 (2H, m, H-16); 1.58 (1H, s, H-9); 1.56 (2H, m, H-1); 1.54 (2H, m, H-22); 1.52 (1H, m, H-20); 1.47 (1H, m, H-6a); 1.43 (2H, m, H-2); 1.31 (1H, m, H-19); 1.29 (1H, m, H-6b); 1.29 (2H, m, H-21); 1.27 (2H, m, H-7); 1.05 (3H, s, H-27); 1.01 (2H, m, H-15); 0.92 (3H, d, J = 6.8 Hz, H-30), 0.90 (3H, s, H-23); 0.87 (3H, s, H-25); 0.82 (3H, d, J = 5.9 Hz, H-29); 0.69 (3H, s, H-26), 0.68 (3H, s, H-24), 0.66 (1H, s, H-5); ^13^C-NMR (DMSO-d_6_) ppm: 178.23 (C-28); 138.18 (C-13); 124.54 (C-12); 76.82 (C-3); 54.77 (C-5); 52.37 (C-18); 47.00 (C-9); 46.81 (C-16); 41.62 (C-14); 39.09 (C-8); 38.48 (C-20); 38.42 (C-19); 38.35 (C-4); 38.22 (C-1); 36.51 (C-10); 36.29 (C-22); 32.69 (C-15); 30.77 (C-7); 28.23 (C-23); 27.52 (C-21); 26.79 (C-2); 23.80 (C-11); 23.24 (C-27); 22.82 (C-16); 21.10 (C-30); 17.97 (C-6); 16.97 (C-29); 16.90 (C-24); 16.03 (C-25); 15.18 (C-26).

### Chromatographis analysis

HPLC was performed using a reversed-phase column (Shimpack CLC-ODS (M) 4.6 mm x 15 cm – particle size 5 μm) eluted at a rate of 0.5 mL/min with an A:B solvent system (A-acetonitrile; B- 1.25% H_3_PO_4 _aqueous; A:B = 86:14 (v/v), with a detection wavelength set at 206 nm and 20 μL injection [14]. To prepare UA standard solution, this compound (7.8 mg) was dissolved in methanol (10 mL) for analysis. Standard solutions were injected (2, 4, 6, 8, 10, and 20 μL respectively) and run for calibration curves. To test plant leaves (5,000 g), appropriate amounts of UA were added to approximately double the contents of this compound in treated materials. The follow-up extractions and HPLC analysis were accomplished in the same manner. The recovery was determined as follows: recovery (%) = (A - B) / C x 100% where, A is the amount of detections, B is the amount of sample without added standard, C is the added amount of the standard. The relative standard deviations (RSD) of recoveries of the UA was 2.1 (n= 5; mean = 98.0).
